# A Systematic Review and Meta-analysis of the Association Between *ACTN3* R577X Genotypes and Performance in Endurance Versus Power Athletes and Non-athletes

**DOI:** 10.1186/s40798-024-00711-x

**Published:** 2024-04-12

**Authors:** El Mokhtar El Ouali, Benjamin Barthelemy, Juan Del Coso, Anthony C. Hackney, Ismail Laher, Karuppasamy Govindasamy, Abdelhalem Mesfioui, Urs Granacher, Hassane Zouhal

**Affiliations:** 1 Laboratory of Biology and Health, Department of Biology, Ibn Tofail University of Kenitra, Kenitra, Morocco; 2 Movement, Sport, Health and Sciences Laboratory (M2S), UFR-STAPS, University of Rennes 2-ENS Cachan, Av. Charles Tillon, 35044 Rennes Cedex, France; 3 Centre for Sport Studies, Rey Juan Carlos University, Fuenlabrada, Spain; 4 University of North Carolina, Chapel Hill, NC USA; 5 Department of Anesthesiology, Pharmacology, and Therapeutics, Faculty of Medicine, University of British Columbia, Vancouver, Canada; 6 Department of Physical Education and Sports Sciences, College of Science and Humanities, SRM Institute of Science and Technology, Kattankulathur, Tamilnadu India; 7 Department of Sport and Sport Science, Exercise and Human Movement Science, University of Freiburg, Freiburg, Germany; 8 Institut International des Sciences du Sport (2IS), 35850 Irodouer, France

**Keywords:** Genetics, Elite athlete, Sports performance, Exercise, Muscle performance

## Abstract

**Background:**

Previous studies reported differences in genotype frequency of the *ACTN3* R577X polymorphisms (rs1815739; RR, RX and XX) in athletes and non-athletic populations. This systematic review with meta-analysis assessed *ACTN3* R577X genotype frequencies in power versus endurance athletes and non-athletes.

**Methods:**

Five electronic databases (PubMed, Web of Science, Scopus, Science Direct, SPORTDiscus) were searched for research articles published until December 31st, 2022. Studies were included if they reported the frequency of the *ACTN3* R577X genotypes in power athletes (e.g., weightlifters) and if they included a comparison with endurance athletes (e.g., long-distance runners) or non-athletic controls. A meta-analysis was then performed using either fixed or random-effects models. Pooled odds ratios (OR) were determined. Heterogeneity was detected using I^2^ and Cochran's Q tests. Publication bias and sensitivity analysis tests were computed.

**Results:**

After screening 476 initial registrations, 25 studies were included in the final analysis (13 different countries; 14,541 participants). In power athletes, the RX genotype was predominant over the two other genotypes: RR versus RX (OR 0.70; 95% CI 0.57–0.85, *p* = 0.0005), RR versus XX (OR 4.26; 95% CI 3.19–5.69, *p* < 0.00001), RX versus XX (OR 6.58; 95% CI 5.66–7.67, *p* < 0.00001). The R allele was higher than the X allele (OR 2.87; 95% CI 2.35–3.50, *p* < 0.00001) in power athletes. Additionally, the frequency of the RR genotype was higher in power athletes than in non-athletes (OR 1.48; 95% CI 1.25–1.75, *p* < 0.00001). The RX genotype was similar in both groups (OR 0.84; 95% CI 0.71–1.00, *p* = 0.06). The XX genotype was lower in power athletes than in controls (OR 0.73; 95% CI 0.64–0.84, *p* < 0.00001). Furthermore, the R allele frequency was higher in power athletes than in controls (OR 1.28; 95% CI 1.19–1.38, *p* < 0.00001). Conversely, a higher frequency of X allele was observed in the control group compared to power athletes (OR 0.78; 95% CI 0.73–0.84, *p* < 0.00001). On the other hand, the frequency of the RR genotype was higher in power athletes than in endurance athletes (OR 1.27; 95% CI 1.09–1.49, *p* = 0.003). The frequency of the RX genotype was similar in both groups (OR 1.07; 95% CI 0.93–1.24, *p* = 0.36). In contrast, the frequency of the XX genotype was lower in power athletes than in endurance athletes (OR 0.63; 95% CI 0.52–0.76, *p* < 0.00001). In addition, the R allele was higher in power athletes than in endurance athletes (OR 1.32; 95% CI 1.11–1.57, *p* = 0.002). However, the X allele was higher in endurance athletes compared to power athletes (OR 0.76; 95% CI 0.64–0.90, *p* = 0.002). Finally, the genotypic and allelic frequency of *ACTN3* genes were similar in male and female power athletes.

**Conclusions:**

The pattern of the frequencies of the *ACTN3* R577X genotypes in power athletes was RX > RR > XX. However, the RR genotype and R allele were overrepresented in power athletes compared to non-athletes and endurance athletes. These data suggest that the RR genotype and R allele, which is associated with a normal expression of α-actinin-3 in fast-twitch muscle fibers, may offer some benefit in improving performance development in muscle strength and power.

**Supplementary Information:**

The online version contains supplementary material available at 10.1186/s40798-024-00711-x.

## Key Points

- The *ACTN3* gene encodes α-actinin-3, a structural protein of Z-lines of skeletal muscle fast-twitch (type II) actin filaments 4,5. Individuals with a ‘null’ XX in the p.R577X polymorphism of this gene are unable to express α-actinin-3, as opposed to individuals with the RR and RX genotypes.
- This systematic review encompasses the meta-analysis of 23 studies that assessed *ACTN3* R577X genotype frequencies in power athletes compared to endurance athletes and non-athletes. The meta-analysis reflected a higher prevalence of RR genotype and R allele in power athletes compared to non-athletes and endurance athletes.
- These outcomes suggest that the RR genotype and the R allele, associated with the full expression of α-actinin-3 in type II muscle fibres may confer advantages for becoming an elite athlete in sports with high demands of muscle strength and power.

**Supplementary Information:**

The online version contains supplementary material available at 10.1186/s40798-024-00711-x.

## Introduction

More than 200 genes and polymorphisms can alter the physical and physiological abilities of athletes [1], and it is estimated that genetic factors account for 40–60% of variations in cardiorespiratory parameters, 50–90% of the variations in anaerobic performance, and 30–70% of variations in muscle strength [2]. Among the many genes that may be related to athletic performance, the R577X polymorphism of the *ACTN3* gene has been suggested to significantly influence sport performance [3].

The *ACTN3* gene encodes α-actinin-3, a structural protein of Z-lines of skeletal muscle fast-twitch (type II) actin filaments [4, 5]. The R577X polymorphism is a single nucleotide polymorphism (SNPs) in the *ACTN3* gene that affects the expression of α-actinin-3 [6]. Specifically, individuals with a ‘null’ 577XX (or simply ‘XX’) genotype are unable to express α-actinin-3, as opposed to individuals with the RR and RX genotypes [7]. The XX genotype does not cause harm, as the lack of α-actinin-3 expression is compensated by the overexpression of α-actinin-2 [7]. In fact, it is estimated that ~ 20% of the world population possess the XX genotype [8]. There is evidence that muscle phenotype may be partly determined by the *ACTN3* R577X polymorphism [9].

The physical fitness qualities speed, power, and muscle strength are supported by the anaerobic metabolism [10]. Several studies investigated the impact of *ACTN3* R577X polymorphisms on anaerobic performance in amateur and professional athletes [11–13], with the general finding of an overrepresentation of the RR genotype (and underrepresentation of the XX genotype) in athletes participating in anaerobic-based disciplines [14]. Specifically, *ACTN3* RR/RX genotypes were associated with better speed, power and muscle strength performances, while the XX genotype was associated with increased endurance [12, 15–18]. Additionally, possessing the *ACTN3* XX genotype has been associated with various phenotypes related to lower anaerobic performance and an increased risk of muscle damage associated with exercise [19–21]. Conversely, although a higher frequency of the *ACTN3* XX genotype was found in endurance athletes in the first study on this topic [21], current evidence suggest no association between the XX genotype of *ACTN3* gene and a higher level of endurance performance [22, 23]. A recent meta-analysis by Tharabenjasin et al. [24] reported a correlation between the R allele and RX genotype of the *ACTN3* polymorphism in elite power athletes. However, the aforementioned meta-analysis did not compare power with endurance athletes in terms of genotypes and alleles of *ACTN3* R577X polymorphism [24]. Furthermore, a significant dominance of the R allele over the X allele was observed (odds ratio [OR] = 1.35, 95% CI 1.18–1.53) in professional soccer players. These findings may suggest an association of the R allele of the ACTN3 gene and better performance in soccer [25].

Studies to date suggest *ACTN3* R577X polymorphisms are strongly associated with anaerobic performance. However, the available studies have not yet been systematically aggregated in the form of a meta-analysis. Here, we aimed to systematically review and meta-analyze studies on *ACTN3* R577X genotype frequencies in power athletes (disciplines of short duration, high intensity and dominated by muscle power) and compared data with endurance athletes (activities of long duration, low to moderate intensity, dominated by endurance and dependent on oxygen availability) and non-athletes. We hypothesized that the RR genotype and R allele would be overrepresented in power athletes compared to non-athletes and endurance athletes [12, 16, 18, 24, 25].

## Methods

The protocol for our review was registered in PROSPERO (registration number: CRD42022360255). The applied systematic searches and meta-analyses were conducted in accordance with the recommendations outlined in the Cochrane Handbook for Systematic Review and Meta-analysis of Interventions [26]. A bibliographic search strategy was performed using the Preferred Reporting Items for Systematic Reviews and Meta-Analyses (PRISMA) Statement [27].

### Working Definitions

Aerobic-based sports (endurance athletes) generally last longer than 5 min and involve an exercise intensity similar to or lower than VO_2max_. Aerobic-based sports are related to the ability of the respiratory and circulatory systems to provide energy and replenish adenosine triphosphate (ATP) through oxidative metabolism [28, 29]. For the purpose of this meta-analysis, studies were included examining road cyclists, marathoners, and long-distance triathletes. In contrast, anaerobic-based sports (power athletes) require an intensity higher than VO_2max_ and energy provision for muscle contraction depend on different metabolic pathways related to the duration of the exercise, including anaerobic glycolysis and the ATP/phosphocreatine system [30]. In the context of this meta-analysis, studies were included with track sprinters, long jumpers, weightlifters.

Elite athletes refer to well-trained athletes who train at least four times per week and compete on an international level.

Regarding the sub-elite athlete category, we refer to athletes who participate in national or state leagues/tournaments and develop the skills necessary to play the sport at a high (national) level, including biomechanics, ball handling skills, and decision-making elements.

Non-athletes and healthy individuals engage in multiple sports and/or types of physical activity in accordance with the World Health Organization (WHO), with at least 150 to 300 min of moderate-intensity activity or 75 to 150 min of vigorous-intensity activity per week and muscle-strengthening activities 2 or more days per week.

### Eligibility Criteria

Only studies that examined the link between the *ACTN3* R577X polymorphism and anaerobic-based exercise performance (e.g., muscle strength, power, speed) were included in our analysis. For this purpose, we selected studies assessing the frequency of the *ACTN3* R577X genotypes in athletes of anaerobic-based sports in which a comparison with either endurance athletes or non-athletic controls was made. We used the following criteria for studies that were included in our systematic review: (1) published in peer-reviewed journals; (2) participants aged 14 years or older; (3) involved athletes of elite or sub-elite level; (4) used validated methods for the characterization of athletes related to anaerobic-based performances (e.g., strength-, power-, or speed-based sports) and contrasted those with endurance athletes (e.g., long-distance runners) or non-athletes; (5) presented data on the frequency of the *ACTN3* R577X polymorphisms (such as RR, RX and XX). Studies were excluded if they (1) did not meet the minimum requirements of an experimental study design (e.g., case reports), (2) did not meet the minimum requirements to classify the sample as athletic; (3) were not written in English; or (4) presented data on the frequency of the *ACTN3* R577X polymorphism only as RR vs X-allele carriers or R-carriers vs XX. Moreover, systematic or narrative review articles were not included in the current systematic review and meta-analysis.

### Literature Search Strategy

Systematic literature searches were conducted in five electronic databases (PubMed, Web of Science, Scopus, Science Direct and SPORTDiscus) from inception until December 31st, 2022, with no restriction on publication dates. The following key terms (and synonyms searched using the MeSH database) were included and combined using the operators “AND”, “OR”, “NOT”: (Alpha-actinin-3 OR *ACTN3*) OR (“*ACTN3* gene” OR “*ACTN3* R577X polymorphism” OR “*ACTN3* R577X” OR “*ACTN3* R577R” OR “*ACTN3* 577XX genotype*” OR “*ACTN3* 577RR genotype*” OR “ACTN3 577RX genotype*”) AND (“anaerobic performance” OR “muscle strength*” OR “muscle power” OR speed OR jump) AND (correlation study OR association OR relationship). In addition, the reference lists and citations (Google Scholar) of the identified studies were explored to detect additional relevant studies.

### Study Selection

The screening and study selection was realized by two investigators (EMEO and BB) using the inclusion and exclusion criteria introduced above. If the title of the article was of potential relevance, the abstract was examined and if still found eligible, the full text was reviewed for evaluation. A third-party consensus meeting was held with a third author (HZ) if the two investigators were unable to reach an agreement on the inclusion of the respective article.

### Data Extraction

Once the inclusion/exclusion criteria were applied, data extraction was conducted to collect information about participants, interventions, comparisons, outcomes and study design (PICOS) in agreement with PRISMA methodology. The following relevant data from each study were extracted: study details (author, year of publication, country), study population (sample size, age, sex), the method used to determine the genotyping of *ACTN3* R577X polymorphism (rs1815739), and the number of participants with RR, RX and XX genotypes in each group. For articles in which these information were not present, associations were calculated using raw data, if available. Two authors (EMEO and BB) extracted data from the included studies, and, a third (HZ) was consulted in case of uncertainty.

### Quality Assessment

The methodological quality of the included studies was assessed using the Physiotherapy Evidence Database (PEDro) scale, which has good reliability and validity [31]. The PEDro scale has eleven possible items and examines external validity (criterion 1) and internal validity (criteria 2–9) of controlled trials and whether there is sufficient statistical information for interpreting results (criteria 10–11). A cut-off threshold of six points on the PEDro scale was used to indicate high-quality studies, as this has been reported to be sufficient to determine the methodological quality level in previous studies. Two independent researchers (EMEO and BB) assessed the quality of the studies, if any disagreements arose, a third researcher (HZ) was contacted, and a unanimous decision was achieved. Any study with a PEDro score below four points would have been excluded from the systematic review and meta-analysis, although none of the studies reviewed were excluded by this criterion.

### Statistical Analyses

To compute associations between the *ACTN3* R577X polymorphism in power athletes compared with endurance athletes and controls (non-athletes), ORs with 95% confidence intervals (CI) and forest plots were calculated using the number of participants with the RR, RX and XX genotype in each study. With these data, we identified the individual and pooled effects of the studies. The degree of heterogeneity between the results of the study was evaluated using the I^2^ test, where I^2^ values of 25%, 50% and 75% were considered as low, medium and high levels of heterogeneity, respectively. The application of fixed or random effects models for each analysis was based on the level of heterogeneity revealed via the I^2^ statistics (< 50% = fixed-effects model; > 50% = random-effects model) and Cochran’s Q [32] test, (significance level at *p* < 0.05). We followed the Cochrane recommendations [33] to detect publication bias in our meta-analysis, and a visual inspection of the funnel plot was performed. Data were analyzed using Cochrane Review Manager (RevMan) version 5.4.1. We performed a one-way ANOVA to calculate the mean percentage distribution and 95% CI for the RR, RX and XX genotypes. Statistical analyses were carried out with GraphPad Prism 9.2.0 (GraphPad Software Inc, San Diego, USA).

## Results

### Selection of Studies and Characteristics of the Included Studies

**Fig. 1:**
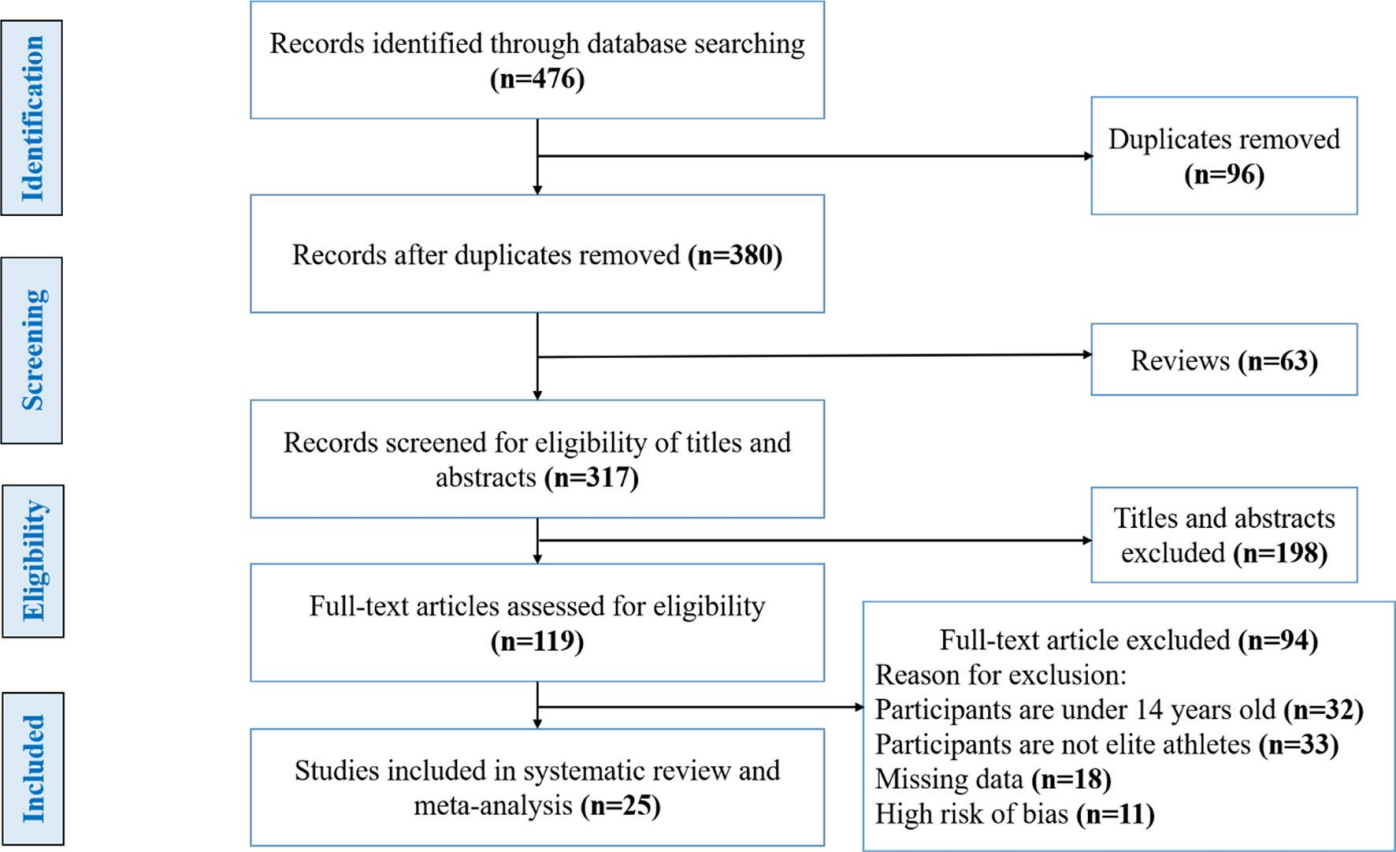
Flowchart of selecting eligible studies for systematic review and meta-analysis

We identified 476 potentially relevant articles, of which 96 studies were duplicates, 63 were review articles and 198 articles were considered ineligible based on inclusion/exclusion criteria and the titles and abstracts (Fig. 1). After reviewing 119 articles, a further 94 were excluded based on the results of the full-text assessment for the following reasons: participants aged under 14 years (n = 32), no elite or sub-elite athletes (n = 33) were included in the study, missing data or uncertain data in one or more genotypes (n = 18) and high risk of bias (n = 11). Based on our inclusion/exclusion criteria and quality assessment, 25 eligible studies were included in our systematic review with meta-analysis [9, 10, 12, 23, 34–54]. These studies were conducted in 13 different countries including Czech Republic, Israel, Brazil, Russia, Spain, Australia, Lithuania, India, Japan, Korea, Italy, Poland and China, with a total sample size of 14,541 participants (7,080 athletes and 7,461 controls). Eleven studies included only males and 14 studies included both male and female participants. The general characteristics of the 25 included studies in this systematic review and meta-analysis were summarized in Table 1.

**Table 1:** Characteristics of studies included in this systematic review with meta-analysis

Study	PEDro scale	Country	Sex	Sample size	Age, years (mean ± SD or range)	Participants and sporting disciplines	Physical test performed	Genotyping
Akazawa et al. [46]	5	Japan	Men and Women	Athletes N: 906 (401F) Controls N: 649 (466F)	Data not shown	Athletes from different sports disciplines Healthy controls	No test performed	RT-PCR
Balkó et al. [34]	5	Czech Republic	Men	N: 15	24.9 ± 6.6	Elite and sub-elite fencers	PCV, PCPB, SCT, HG D, RPT/30s and Pmax	Data not shown
Ben-Zaken et al. [35]	5	Israel	Men and Women	N: 211 CG: 86 (31F) SJ: 71 (24F) WL: 54 (13F)	25.8 ± 3.7 24.8 ± 6.1 28.7 ± 9.4	Sprinters and long jumpers, weightlifters, non-athletic control	SJ: 100m run (sprinters) Long jump (jumpers) WL: Snatch Clean and jerk	RT-PCR
Coelho et al. [36]	5	Brazil	Men	N: 138 U17: 32 U20: 38 Prof: 68	17.3 ± 5.3 20.6 ± 3.6 23.2 ± 6.4	Under-17, Under-20 and professionals of a Brazilian first division soccer team	V10, V20, V30, SJ, CMJ and VO_2max_	PCR–RFLP
Druzhevskaya et al. [47]	5	Russia	Men and Women	Athletes N: 486 (123F) Controls N: 1197 (673F)	RR: 24 ± 0.7 RX: 24.5 ± 0.6 XX: 24.1 ± 1.0 RR: 17.1 ± 0.2 RX: 17.2 ± 0.2 XX: 16.7 ± 0.4	Highly elite, elite, sub-elite, average and controls (healthy subjects)	No test performed	PCR–RFLP
Eynon et al. [23]	6	Israel	Men and Women	Sprinters N: 81 (22F) Controls N: 240	31.4 ± 14.2	Sprinters (top-level/national-level) and healthy individuals	No test performed	PCR–RFLP
Eynon et al. [37]	6	Israel	Men and Women	Athletes N: 155 (36F) Controls N: 240 (73F)	35.9 ± 12.2	Track and field athletes (elite level/national-level) and healthy individuals	No test performed	PCR–RFLP
Eynon et al. [38]	5	Spain	Men	Athletes N: 633 SP: 273 PP: 217 RP: 143 Controls N: 808 SP: 343 PP: 354 RP: 111	SP(PA): 20–33 SP(EA): 20–39 SP(C): 19–32 PP(C): 19–32	Endurance/power athletes and healthy individuals	No test performed	RT-PCR
Eynon et al. [39]	5	Australia	Men	Athletes N: 888 SP: 323 PP: 341 RP: 224 Controls N: 568 SP: 103 PP: 354 RP: 111	SP(EA): 20–39 SP(C): 19–32 PP(Hoc): 28 ± 4 PP(Ha): 26 ± 2 PP(soc): 26 ± 6 PP(C): 19–32 RP(C): 19–32	Team-sport, sprint/power and endurance athletes and healthy individuals	No test performed	RT-PCR
Garatachea et al. [48]	5	Spain	Men and Women	Athletes N: 102 (41F) Controls N: 283 (67F)	25 ± 5.4 21.1 ± 2	Healthy young adults and elite basketball players	SJ CMJ	PCR–RFLP
Gineviciene et al. [40]	5	Lithuania	Men and Women	Athletes N: 161 (33F) Controls N: 1202 (662F)	23 ± 6.5 29 ± 8.5	Professional strength/power athletes and healthy individuals	No test performed	PCR–RFLP
Grover et al. [41]	3	India	Men	Athletes N: 23 Controls N: 25	Data not shown	Elite power/speed athletes and non-athletes	No test performed	PCR–RFLP
Kikuchi et al. [49]	5	Japan	Men and women	N:1057 PS: 627 EA: 430 Controls N: 810	Data not shown	Regional, National and International PS and EA Nonathletic individuals	No test performed	RT-PCR
Kim et al. [42]	5	Korea	Men and women	N: 975 ST: 63 PS: 58 Controls N: 854	22.2 ± 3.6 20.8 ± 4.6 32.6 ± 4.8	Running, speed, skating, swimming, and weightlifting athletes Non-athletes	No test performed	RT-PCR
Massidda et al. [10]	5	Italy	Men	N: 178 TS: 74 PS: 64 EA: 40 Controls N: 190	Data not shown	Elite ST, PS and EA Healthy individuals	No test performed	PCR–RFLP
Massidda et al. [54]	5	Italy	Men and women	Athletes N: 35 (18F) Controls N: 53 (22F)	Data not shown	Junior and senior artistic gymnastics team	No test performed	PCR–RFLP
Melián Ortiz et al. [52]	5	Spain	Men and women	N: 80 CMW: 20 EMW: 20 C-EMW: 20 IMW: 20	26.7 ± 2.29 22.6 ± 3.4 28.1 ± 9 22.3 ± 3.1	Healthy population	LJ Sargent Test Power jump (Sayer equation) Sprint test	RT- PCR
Orysiak et al. [9]	5	Poland	Men	N: 200	Ca: 18.1 ± 1.6 I hoc: 17.5 ± 0.9 SW: 15.1 ± 1.5 VB: 17.2 ± 0.7	Elite canoeing, ice hockey players, swimming, and volleyball players	ACMJ CMJ SPJ	PCR–RFLP
Orysiak et al. [50]	4	Poland	Men and Women	N: 398 (132F)	M: 16.7 ± 2.1 F: 15.8 ± 2.0	Elite canoeing, ice hockey players, swimming, and volleyball players	CMJ SPJ Muscle Strength	PCR–RFLP
Papadimitriou et al. [53]	6	Australia	Men	N: 555	Data not shown	100m, 200m and 400m sprinters	No test performed	RT- PCR
Pasqualetti et al. [43]	5	Italy	Men	N: 27	22.6 ± 2.9	Rugby elite players	505-test 20m sprint test RSA YOYO IRT1 CMJ	PCR–RFLP
Petr et al. [44]	5	Czech Republic	Men	N: 99	25.4 ± 4.5	Professional soccer players	CMJ with and without hands on the waist SJ Knee extensors and flexors strength	PCR–RFLP
Pimenta et al. [45]	5	Brazil	Men	N: 200	24.4 ± 2	Professional soccer players	10m, 20m and 30m sprint test SJ and CMJ YOYO IRT1	PCR–RFLP
Wenjia Chen et al. [51]	6	China	Men and Women	N: 314 (53 F) Controls N: 206 (88 F)	MS: (10–10.25)s: 25.2 ± 3.4 (10.26–10.50)s: 19.2 ± 1.9 (10.51–10.93)s: 18.9 ± 1.1 (10.94–11.74)s: 15.3 ± 1.2 FS: (11.60–11.70)s: 22.6 ± 5.1 (11.71–12.33)s: 19.6 ± 3.8 (12.34–13.04)s: 15.2 ± 0.9 CM: 20.3 ± 1.2 CF: 19.9 ± 1.4	Elite sprinters Healthy controls	100m sprint, standing jump and standing triple jump	PCR–RFLP
Yang et al. [12]	5	China	Men and Women	N: 153 Athletes: 103 CG: 50	24.3 ± 3.2 (20–35) 25.5 ± 3.0 (20–30)	Elite EA and PS athletes Healthy CG	Standing Long Jump Standing Vertical Jump	RT-PCR

Hoc, hockey; Han, handball; Soc, soccer; Ca, canoe; I Hoc, Ice Hockey; SW, swimming; VB, volleyball; MS, male sprinters; FS, female sprinters; CM, control male; CF, control female; PCV, lunge movement times; PCPB, direct lunge movement time; SCT, specific shuttle test; HG D, dominant limb handgrip; RPT/30 s, maximum number of revolutions in 30 s during Wingate test; Pmax, maximum value in Wingate test; SP, Spanish population; PP, polish population; RP, Russian population; PCR–RFLP, polymerase chain reaction-restriction fragment length polymorphism; PS, Sprint/Power Athletes; EA, Endurance Athletes; ST, Strength Athletes; CG, Control Group; TS, Team Sport; CMW, Concentric muscle work; EMW, Eccentric muscle work; C-EMW, Concentric-eccentric muscle work; IMW: Isometric muscle work; ACMJ: Akimbo countermovement jumps; SPJ, Spike jumps; LJ, Long jump; RSA: Repeated-sprint ability; YOYO IRT1, Yo-yo intermittent recovery test level 1

### Study Quality Assessment

The eligible studies had satisfactory methodological quality, as summarized in Table 2. We used the PEDro scale to identify one low quality study (4 points), 21 studies were of moderate quality (5 or 6 points) and three studies were of high quality (7 points or more).

**Table 2:** Physiotherapy evidence database (PEDro) score of the included longitudinal studies

Study	Assessment criteria											PEDro scale	Quality
	1	2	3	4	5	6	7	8	9	10	11		
Akazawa et al. [46]	1	0	0	1	0	0	0	1	1	1	0	5	Medium
Balkó et al. [34]	1	0	0	1	0	0	0	1	1	1	1	6	Medium
Ben-Zaken et al. [35]	1	0	0	1	0	0	0	1	1	1	1	6	Medium
Coelho et al. [36]	1	0	0	1	0	0	0	1	1	1	1	6	Medium
Druzhevskaya et al. [47]	1	0	0	1	0	0	0	1	1	1	1	6	Medium
Eynon et al. [23]	1	1	0	1	0	0	0	1	1	1	1	7	High
Eynon et al. [37]	1	1	0	1	0	0	0	1	1	1	1	7	High
Eynon et al. [38]	1	0	0	1	0	0	0	1	1	1	1	6	Medium
Eynon et al. [39]	1	0	0	1	0	0	0	1	1	1	1	6	Medium
Garatachea et al. [48]	1	0	0	1	0	0	0	1	1	1	1	6	Medium
Gineviciene et al. [40]	1	0	0	1	0	0	0	1	1	1	1	6	Medium
Grover et al. [41]	0	0	0	1	0	0	0	1	1	0	1	4	Low
Kikuchi et al. [49]	1	0	0	1	0	0	0	1	1	1	1	6	Medium
Kim et al. [42]	1	0	0	1	0	0	0	1	1	1	1	6	Medium
Massidda et al. [10]	1	0	0	1	0	0	0	1	1	1	1	6	Medium
Massidda et al. [54]	1	0	0	1	0	0	0	1	1	1	0	5	Medium
Melián Ortiz et al. [52]	1	0	0	1	0	0	0	1	1	1	1	6	Medium
Orysiak et al. [9]	1	0	0	1	0	0	0	1	1	1	1	6	Medium
Orysiak et al. [50]	1	0	0	1	0	0	0	1	1	0	1	5	Medium
Papadimitriou et al. [53]	1	0	0	1	0	0	0	1	1	1	1	6	Medium
Pasqualetti et al. [43]	1	0	0	1	0	0	0	1	1	1	1	6	Medium
Petr et al. [44]	1	0	0	1	0	0	0	1	1	1	1	6	Medium
Pimenta et al. [45]	1	0	0	1	0	0	0	1	1	1	1	6	Medium
Wenjia Chen et al. [51]	1	1		1	0	0	0	1	1	1	1	7	High
Yang et al. [12]	1	0	0	1	0	0	0	1	1	1	1	6	Medium

### Meta-Regression and Cumulative Meta-analysis

For power athletes, our search identified 19 eligible studies [9, 10, 12, 23, 34–46, 52, 53] with 2,212 athletes genotyped the *ACTN3* R577X polymorphism. Another 15 studies [9, 10, 12, 23, 35–42, 44, 46, 46, 49] were identified with power athletes (n = 2310) and controls (n = 6203) genotyped the *ACTN3* R577X polymorphism. Eight studies [9, 10, 12, 37–39, 46, 49] were included with power athletes (n = 1722) and endurance athletes (n = 1357) genotyped the *ACTN3* R577X polymorphism In addition, five studies compared *ACTN3* R577X genotypes in male (n = 564) and female (n = 296) power athletes [47, 48, 50, 51, 54].

These studies included fencers, track sprinters, long jumpers, weightlifters, football players, basketball players, speed skaters, sprint swimmers, ice hockey players, volleyball players, rugby players, sprint canoers, wrestlers, gymnasts, mixed samples of athletes (such as jumpers, sprinters and decathlon athletes) or samples with unspecified power-based sports. All of these sport disciplines were classified as muscle power dominated in the context of this meta-analyses. Additionally, the samples including long-distance runners, marathoners, road cyclists, endurance rowers, and long-distance triathletes, and the samples with unspecified endurance-based sports were categorized as endurance dominated for the purpose of this meta-analysis.

#### ACTN3 R577X Polymorphism in Power Athletes

**Fig. 2:**
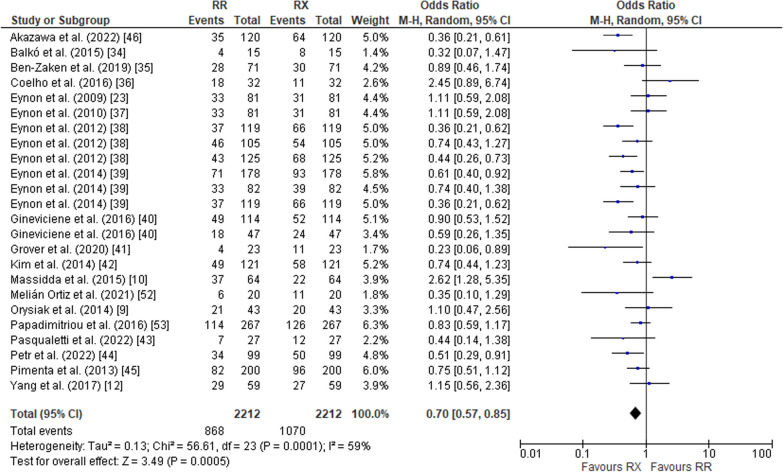
Forest plot of ACTN3 R577X polymorphism in power athletes (RR vs. RX genotypes) [9, 10, 12, 23, 34–46, 52, 53]

**Fig. 3:**
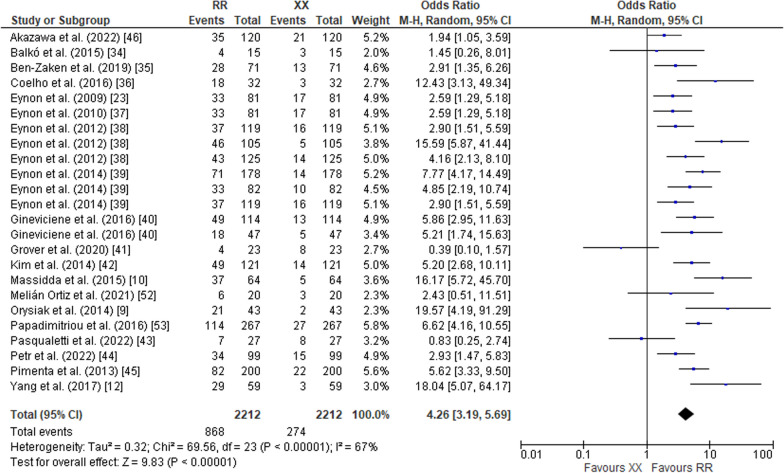
Forest plot of ACTN3 R577X polymorphism in power athletes (RR vs. XX genotypes) [9, 10, 12, 23, 34–46, 52, 53]

**Fig. 4:**
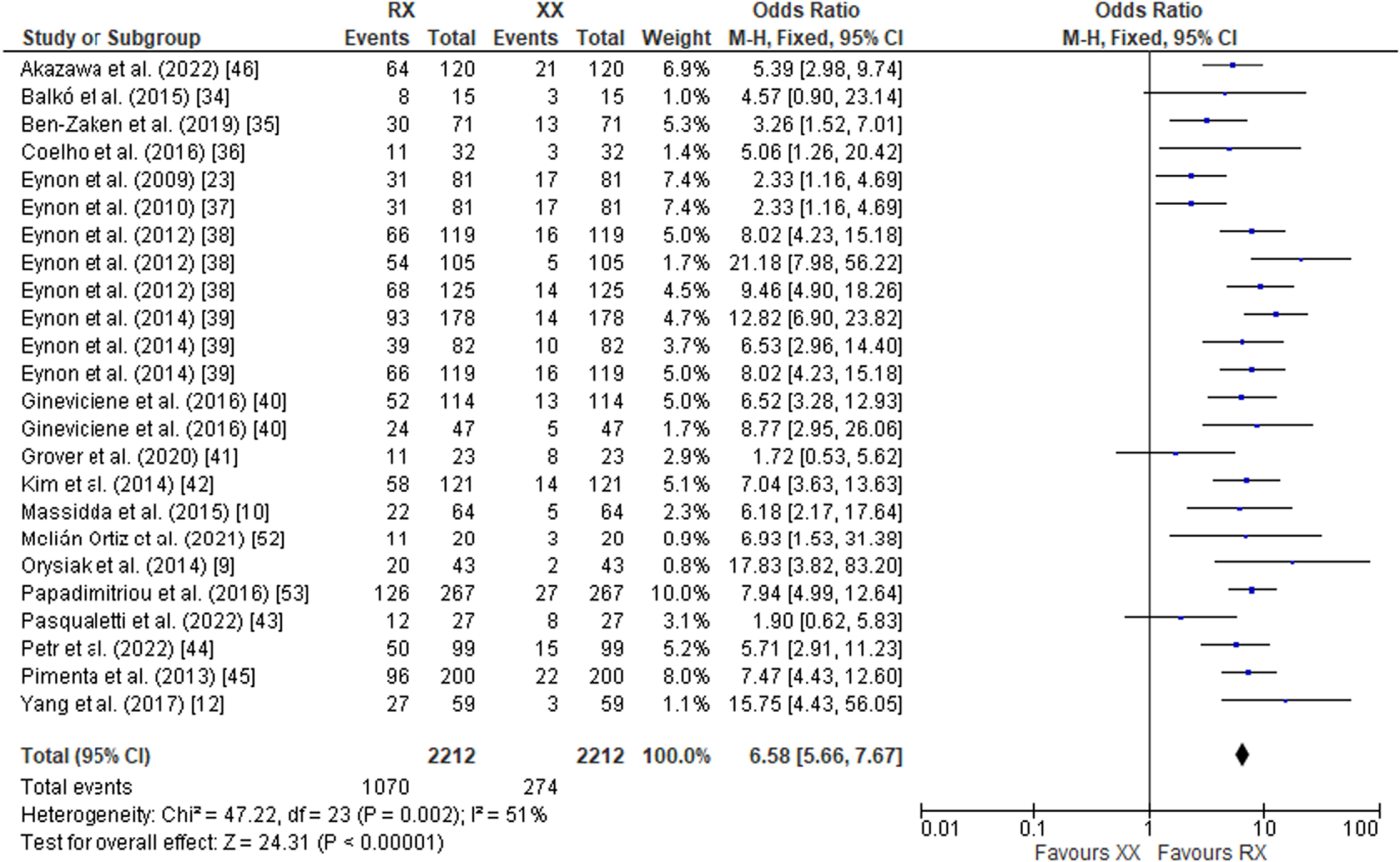
Forest plot of ACTN3 R577X polymorphism in power athletes (RX vs. XX genotypes) [9, 10, 12, 23, 34–46, 52, 53]

**Fig. 5:**
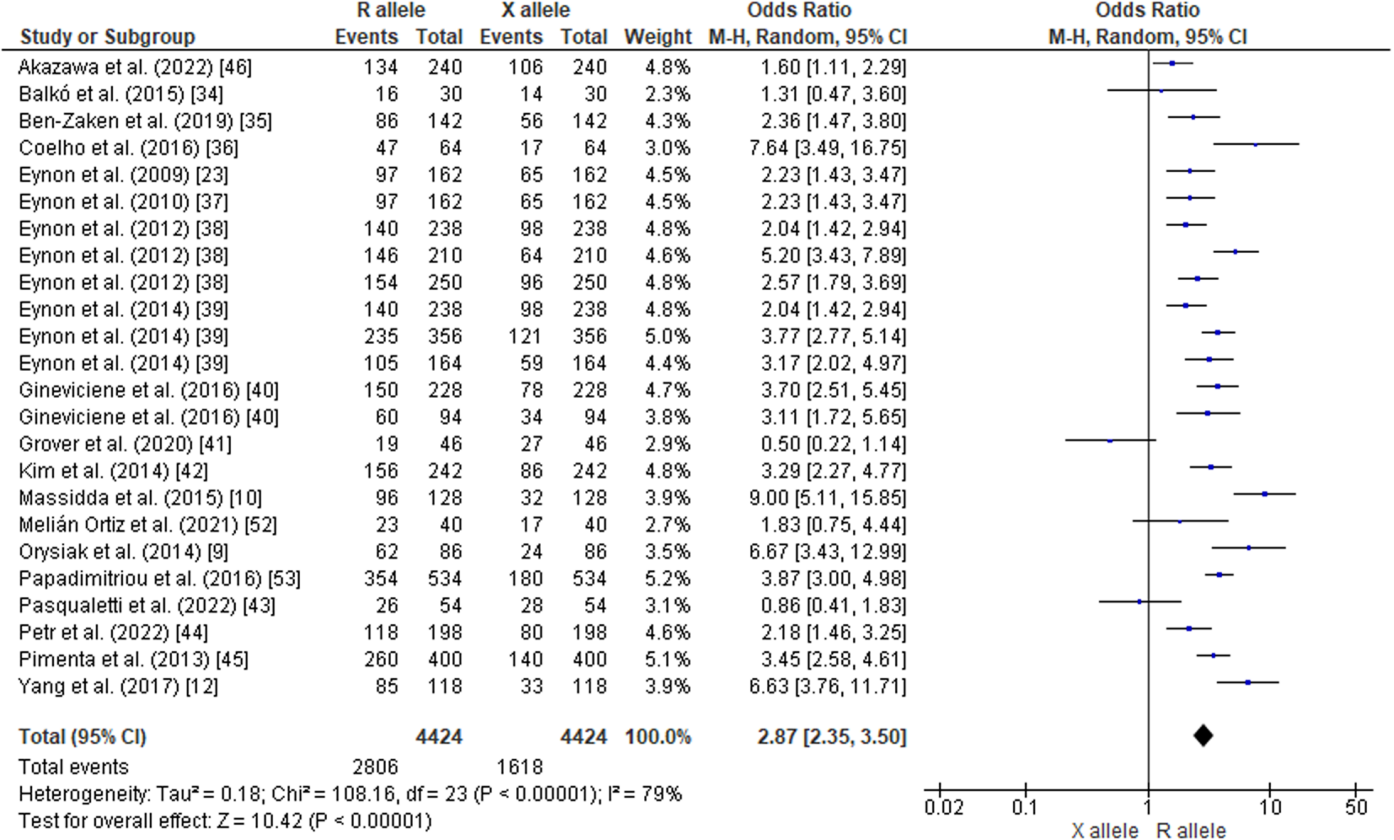
Forest plot of ACTN3 R577X polymorphism in power athletes (R vs. X alleles) [9, 10, 12, 23, 34–46, 52, 53]

The pooled data on studies of power athletes showed a lower frequency of the RR genotype compared to the RX genotype (OR 0.70; 95% CI 0.57–0.85, *p* = 0.0005) with a moderate heterogeneity of 59% (Fig. 2). The pooled data also indicated a higher frequency of RR in power athletes compared to XX counterparts (OR 4.26; 95% CI 3.19–5.69, *p* < 0.00001) with a moderate heterogeneity of 67% (Fig. 3). Additionally, the frequency of RX in power athletes was higher than XX counterparts (OR 6.58, 95% CI 5.66–7.67, *p* < 0.00001) with a moderate heterogeneity of 51% (Fig. 4). The pooled data showed a higher frequency of the R allele compared to the X allele (OR 2.87; 95% CI 2.35–3.50, *p* < 0.00001, 79% heterogeneity) (Fig. 5).

#### ACTN3 R577X Polymorphism in Power Athletes Compared to Controls

**Fig. 6:**
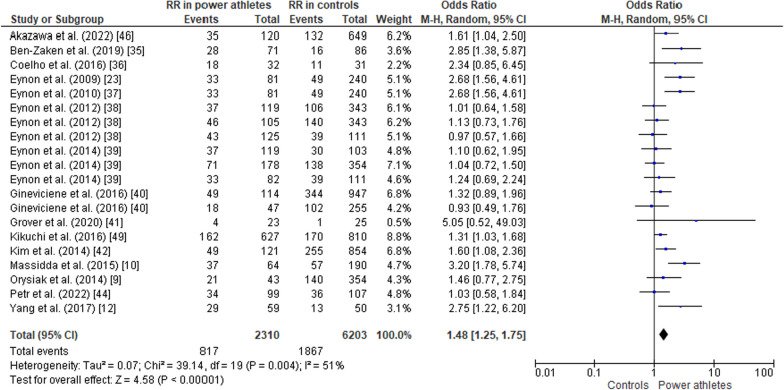
Forest plot of RR genotype expression in power athletes versus controls [9, 10, 12, 23, 34–42, 44, 46]

**Fig. 7:**
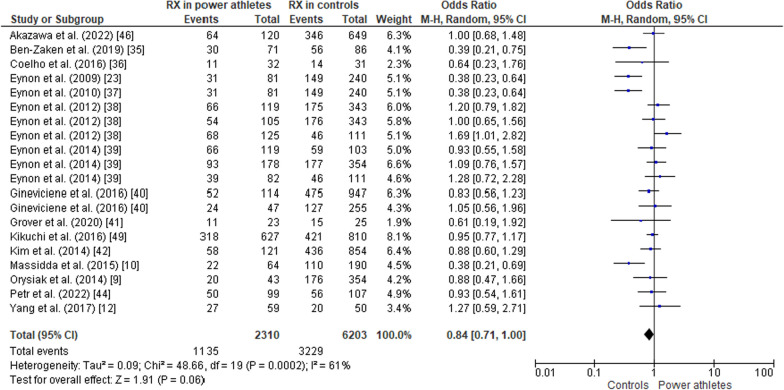
Forest plot of RX genotype expression in power athletes versus controls [9, 10, 12, 23, 34–42, 44, 46]

**Fig. 8:**
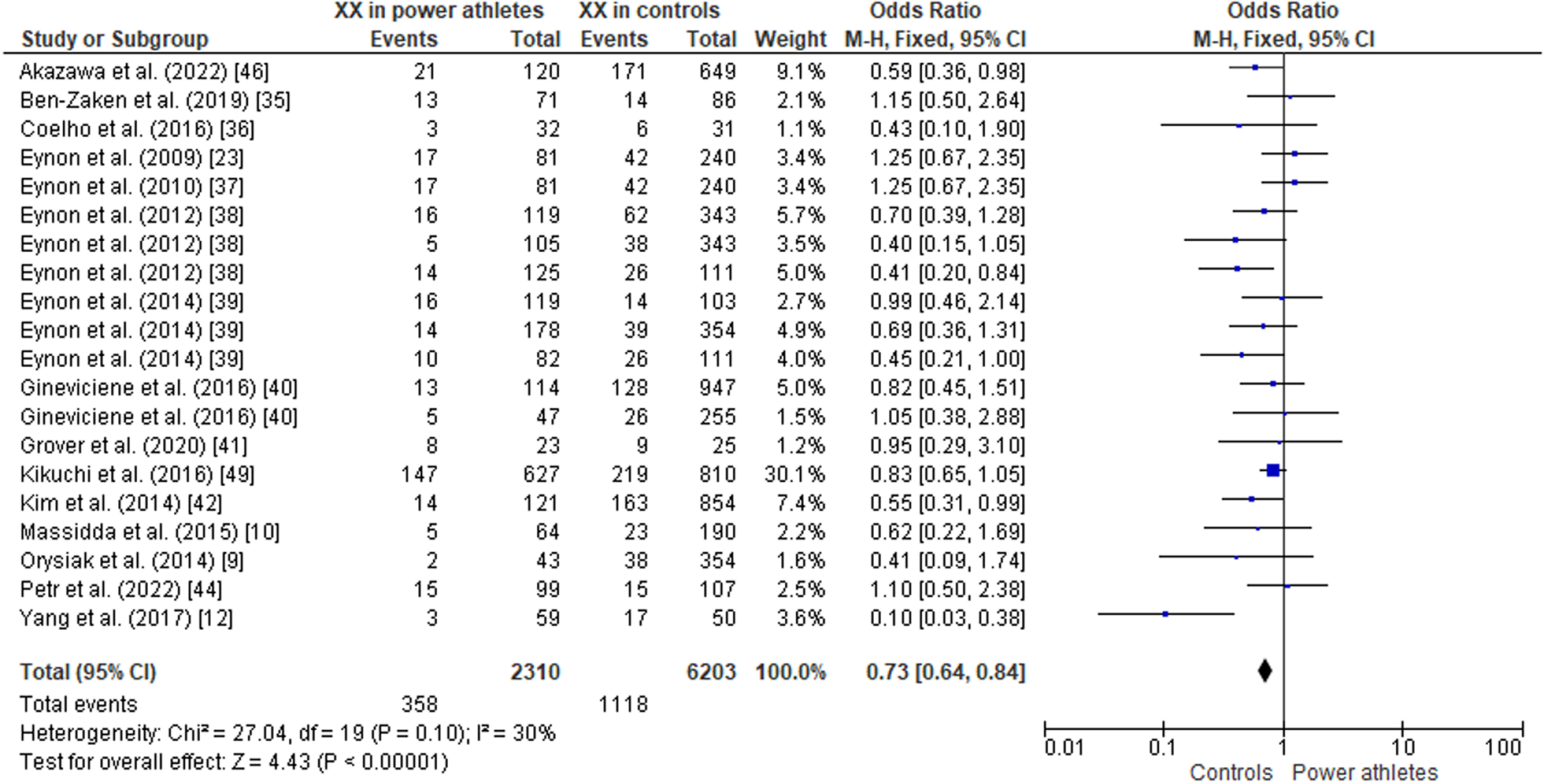
Forest plot of XX genotype expression in power athletes versus controls [9, 10, 12, 23, 34–42, 44, 46]

**Fig. 9:**
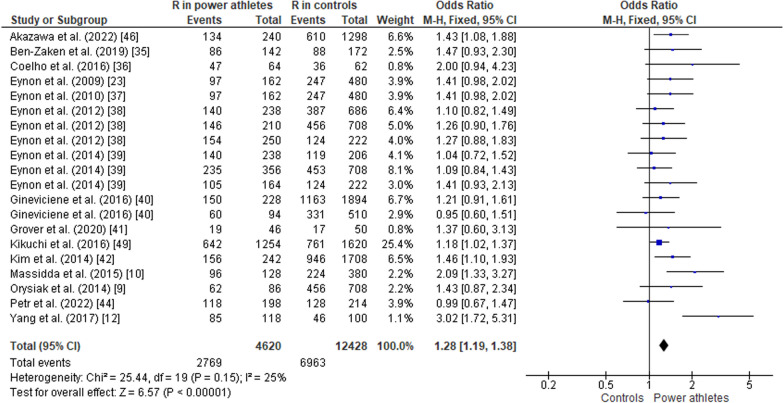
Forest plot of R allele in power athletes versus controls [9, 10, 12, 23, 34–42, 44, 46]

**Fig. 10:**
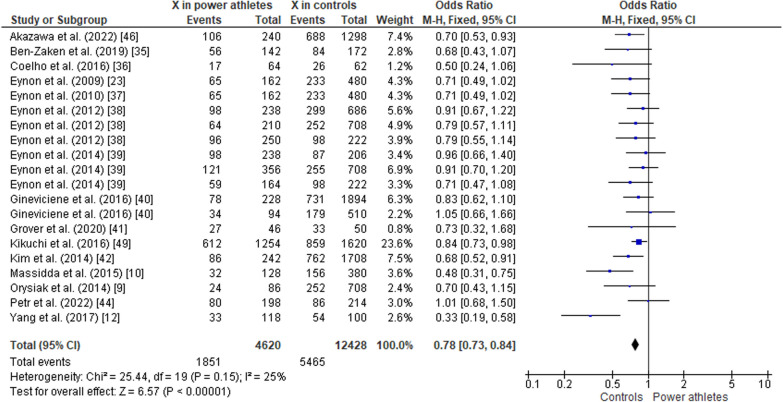
Forest plot of X allele in power athletes versus controls [9, 10, 12, 23, 34–42, 44, 46]

The pooled data comparing ACTN3 R577X polymorphism in power athletes versus controls (non-athletes) showed a greater frequency of the RR genotype in athletes than in controls (OR 1.48, 95% CI 1.25–1.75, *p* < 0.00001, moderate heterogeneity of 51%) (Fig. 6). The frequency of the RX genotype was similar in both groups (OR 0.84, 95% CI 0.71–1.00, *p* = 0.06, moderate heterogeneity of 61%) (Fig. 7). In contrast, the frequency of the XX genotype was lower in power athletes than in controls (OR 0.73, 95% CI 0.64–0.84, *p* < 0.00001, low heterogeneity of 30%) (Fig. 8). Furthermore, there was higher frequency of the R allele in power athletes compared to controls (OR 1.28; 95% CI 1.19–1.38; *p* < 0.00001) with 25% heterogeneity (Fig. 9). Conversely, a high frequency of the X allele was observed in the control group compared to power athletes (OR 0.78; 95% CI 0.73–0.84, *p* < 0.00001) with 25% heterogeneity (Fig. 10).

#### ACTN3 R577X Polymorphism in Power Athletes Compared to Endurance Athletes

**Fig. 11:**
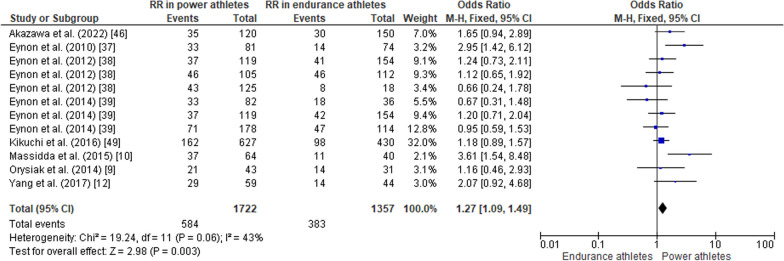
Forest plot of RR genotype expression in power versus endurance athletes [9, 10, 12, 37–39, 46, 49]

**Fig. 12:**
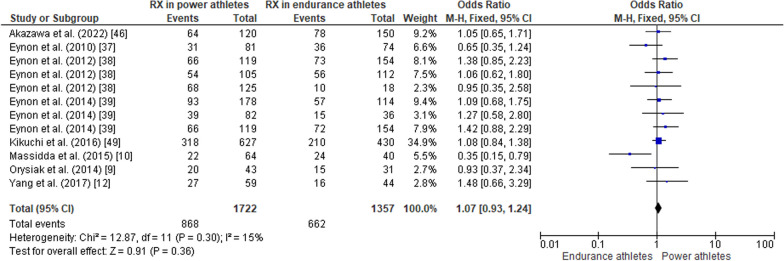
Forest plot of RX genotype expression in power versus endurance athletes [9, 10, 12, 37–39, 46, 49]

**Fig. 13:**
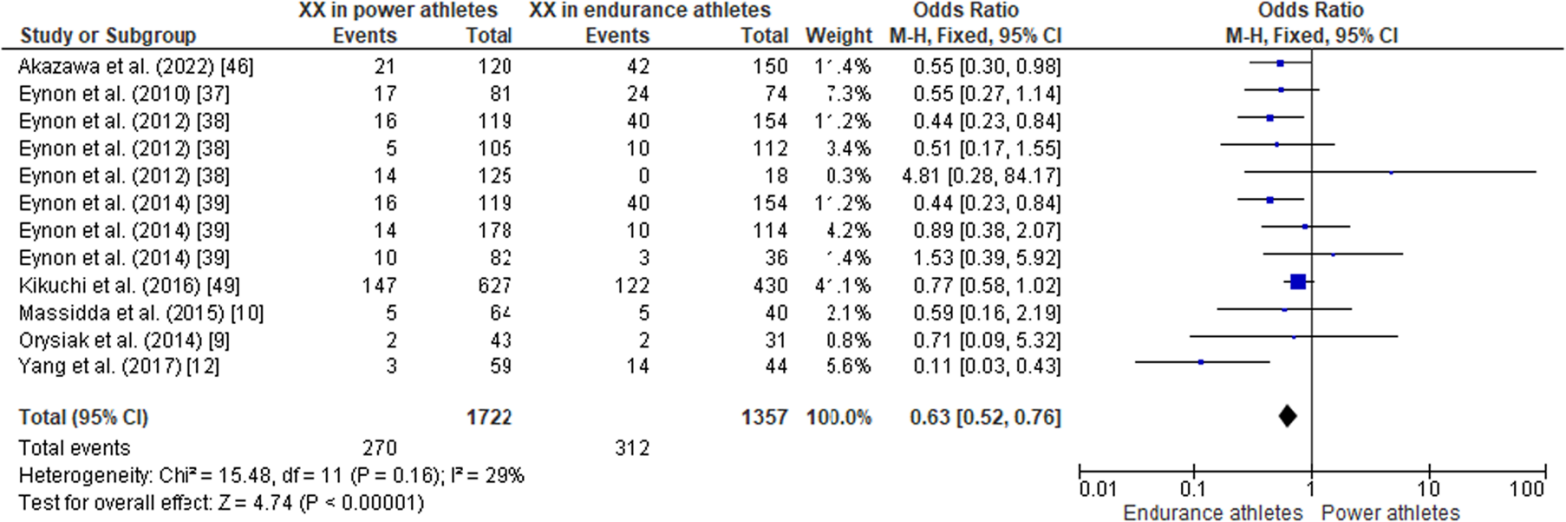
Forest plot of XX genotype expression in power versus endurance athletes [9, 10, 12, 37–39, 46, 49]

**Fig. 14:**
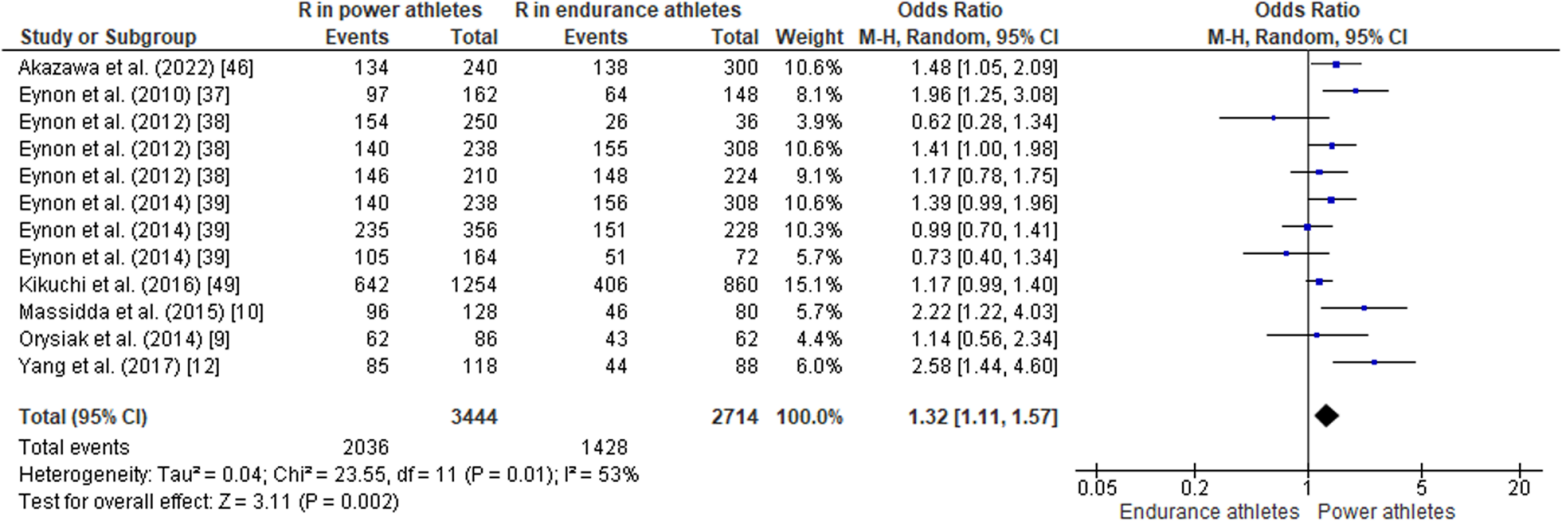
Forest plot of R allele in power athletes versus endurance athletes [9, 10, 12, 37–39, 46, 49]

**Fig. 15:**
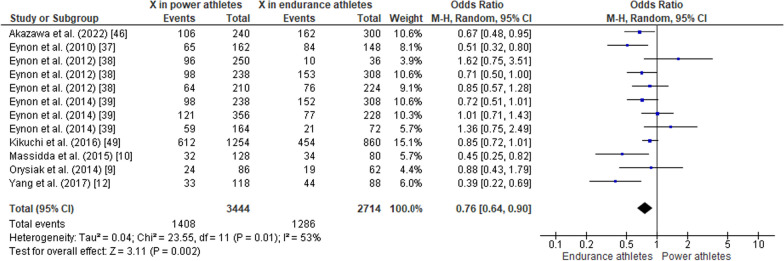
Forest plot of X allele in power athletes versus endurance athletes [9, 10, 12, 37–39, 46, 49]

When comparing the *ACTN3* R577X polymorphism in power athletes versus endurance athletes, the pooled data indicated that the frequency of the RR genotype was higher in power athletes than in endurance athletes (OR 1.27, 95% CI 1.09–1.49, *p* = 0.003, low heterogeneity of 43%) (Fig. 11), while no significant differences were observed between groups of athletes for the frequency of the RX genotype (OR 1.07, 95% CI 0.93–1.24, *p* = 0.36, low heterogeneity of 15%) (Fig. 12). The frequency of the XX genotype in power athletes was lower than in endurance athletes (OR 0.63, 95% CI 0.52–0.76, *p* < 0.00001, low heterogeneity of 29%) (Fig. 13). In addition, a high frequency of the R allele occurred in power athletes compared to endurance athletes (OR 1.32; 95% CI 1.11–1.57, *p* = 0.002) with 53% of heterogeneity (Fig. 14). Conversely, the frequency of allele X was higher in endurance athletes than in power athletes (OR 0.76; 95% CI 0.64–0.90, *p* = 0.002) with a heterogeneity of 53% (Fig. 15).

#### ACTN3 R577X Polymorphism in Male and Female Power Athletes

**Fig. 16:**
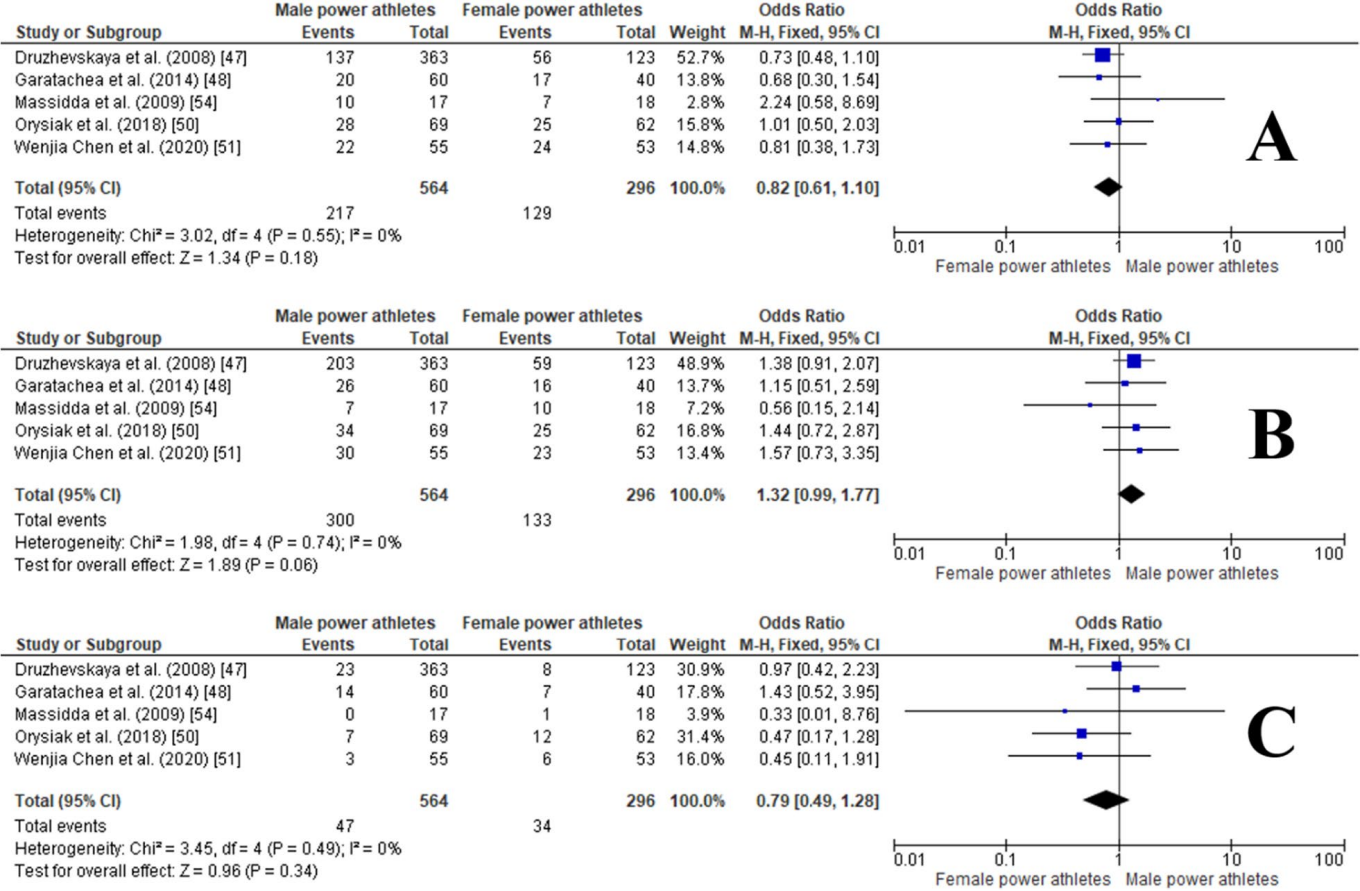
Forest plot of ACTN3 R577X polymorphism in male versus female power athletes. RR (**A**), RX (**B**) and XX (**C**) genotypes [47, 48, 50, 51, 54]

**Fig. 17:**
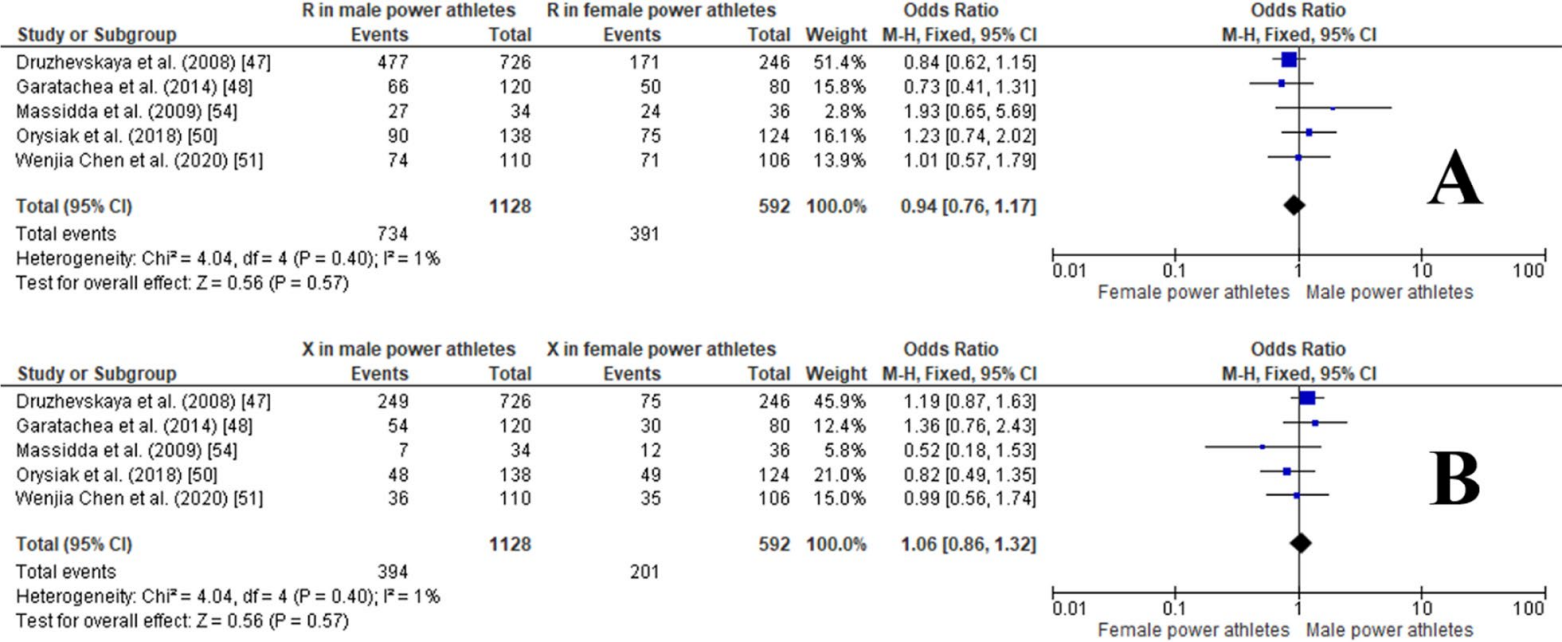
Forest plot of R (**A**) and X alleles (**B**) frequencies in male vs. female power athletes [47, 48, 50, 51, 54]

The pooled data indicated that the frequency of the RR genotype was similar in males versus female power athletes (OR 0.82, 95% CI 0.61–1.10, *p* = 0.18, 0% of heterogeneity*)* (Fig. 16A), while the RX genotype was slightly more frequent in male than in female power athletes, although this was not statistically significant (OR1.32, 95% CI 0.99–1.77, *p* = 0.06, 0% heterogeneity) (Fig. 16B). The frequencies of the XX genotype were similar in males and females power athletes (OR 0.79, 95% CI 0.49–1.28, *p* = 0.34, 0% heterogeneity) (Fig. 16C). Moreover, no significant differences were observed in the frequencies of R and X alleles in male (OR 0.94, 95% CI of 0.76–1.17, *p* = 0.57, 1% of heterogeneity) (Fig. 17A) and female power athletes (OR 1.06, 95% CI 0.86–1.32, *p* = 0.57, 1% heterogeneity) (Fig. 17B).

#### Genotypic Frequency of ACTN3 R577X Polymorphism in all Groups

**Fig. 18:**
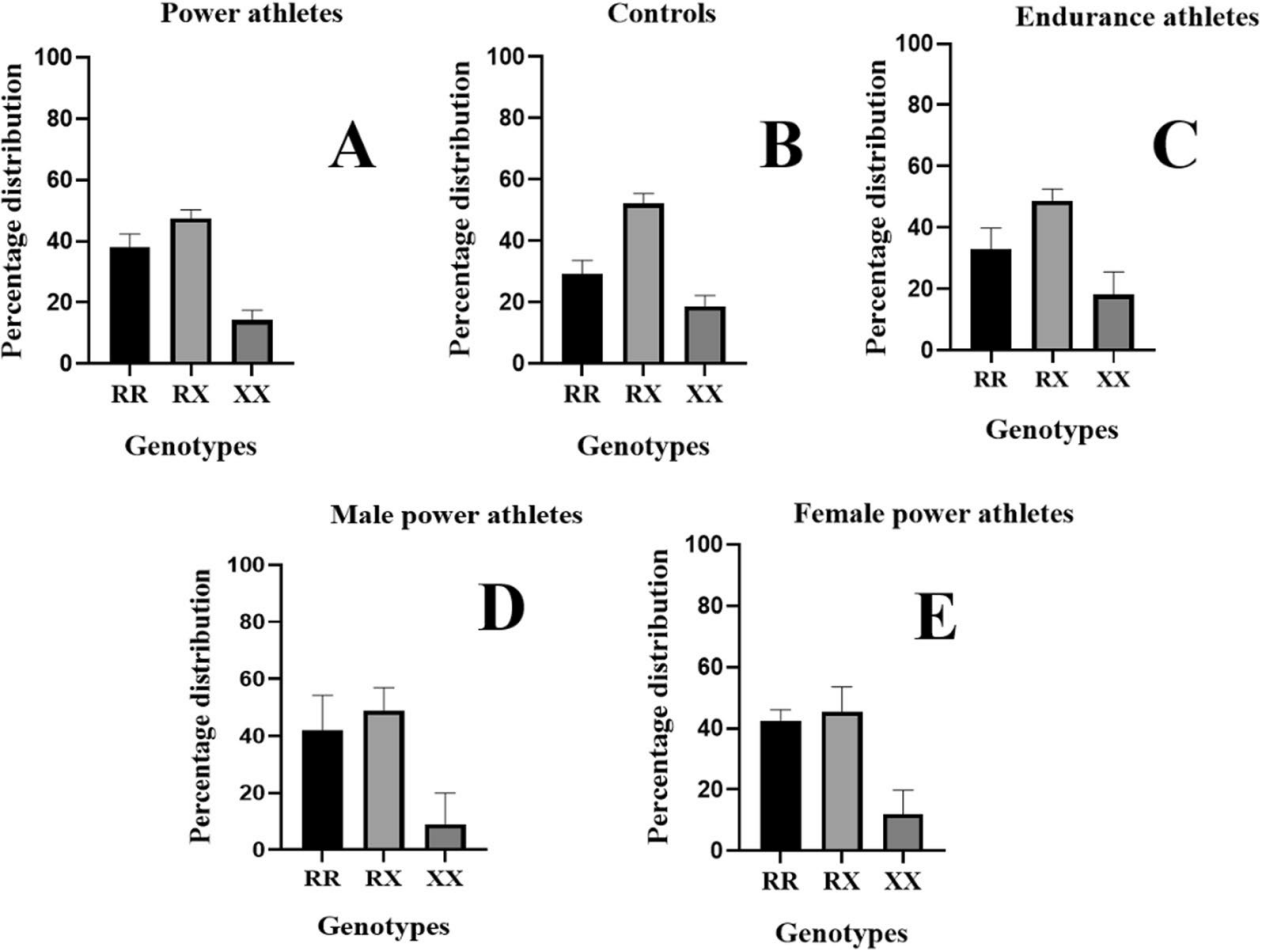
Percentage distribution of RR, RX and XX genotypes in the different groups included in this meta-analysis. Power athletes (**A**) [9, 10, 12, 23, 34–46, 52, 53], controls (**B**) [9, 10, 12, 23, 34–42, 44, 46], endurance athletes (**C**) [9, 10, 12, 37–39, 46, 49], male power athletes (**D**) [47, 48, 50, 51, 54] and female power athletes (**E**) [47, 48, 50, 51, 54]

Subsequently, we performed further analyses by calculating the mean percentage distribution and determined the 95% CI for RR, RX, and XX genotypes in the various groups included in this meta-analysis. The main objective was to identify the dominant genotype in power and endurance athletes as well as in controls (non-athletes). The results of this analysis are presented in Table 3 and represented visually in Fig. 18.

**Table 3:** Percentage distribution of RR, RX and XX genotypes in the different groups. Values are means and 95% CI

Groups	Mean	Lower 95% CI	Upper 95% CI
*Power athletes*			
RR	38.44	34.45	42.43
RX	47.50	44.84	50.17
XX	14.05	10.93	17.18
*Endurance athletes*			
RR	33.07	26.22	39.91
RX	48.80	44.99	52.61
XX	18.13	10.75	25.50
*Controls*			
RR	29.22	24.82	33.62
RX	52.09	48.80	55.38
XX	18.65	15.09	22.20
*Male power athletes*			
RR	42.09	29.96	54.23
RX	48.85	40.71	56.99
XX	9.05	− 1.83	19.94
*Female power athletes*			
RR	42.50	38.85	46.16
RX	45.45	37.38	53.52
XX	12.05	4.28	19.81

### Sensitivity Analyses

We used both the fixed-effects model and a random-effects model based on the percentage of heterogeneity in our initial sensitivity analysis. As the use of fixed-effects or random-effects independently of heterogeneity reflects the same between-groups differences for the comparison of populations, we next conducted another sensitivity analysis by removing one study at a time to determine the impact of the deleted data on the ORs and heterogeneity. One study [49] was excluded from the meta-analysis due to excessive heterogeneity caused by a large sample size. The corresponding OR for the genotype comparisons did not change with this additional sensitivity analysis, indicating the robustness of our data.

### Publication Bias

A visual inspection of the funnel plots for each meta-analysis indicated no publication bias for any of the meta-analyses included in this investigation. The funnel plots for the comparison of genotype frequencies are included in the Additional file 1.

## Discussion

This systematic review with meta-analysis assessed *ACTN3* R577X genotype frequencies in power athletes compared with populations of endurance athletes and non-athletes. Our meta-analysis showed that the frequency of *ACTN3* R577X genotypes in power athletes followed an RX > RR > XX pattern, which is similar to that in non-athletes and endurance athletes. However, the RR genotype was over-represented in power athletes compared to non-athletes and endurance athletes. In contrast, the XX genotype was under-represented in power athletes compared to non-athletes and endurance athletes. Additionally, the RX genotype was slightly more frequent in male than in female power athletes, but this was not statistically significant. Collectively, these data demonstrate the frequency of the ACTN3 R577X genotype in elite and sub-elite strength athletes, which is characterized by a higher proportion of RR athletes and a lower prevalence of XX genotype. The genotype *ACTN3* R577X affects the expression of α-actinin-3 in fast-twitch fibres [7], suggesting that a full expression of α-actinin-3 associated with the RR genotype may increase the likelihood of reaching the status of elite athletes in anaerobic sporting events.

### ACTN3 R577X Polymorphism in Power Athletes Compared to Controls

Our meta-analysis indicates that the RR genotype and the R allele are more frequent in power athletes than in controls, which is supported by other reports [24, 35, 45, 48]. Thus, we provide additional evidence for the important role of the RR genotype in performance in speed/power sports. Studies of R and X alleles in athletes and controls and in athletes of different sport types (mainly power-based versus endurance-based) suggest that the R allele is more frequent in power athletes than in control (non-athletes) [23, 38] or endurance athletes [55]. Other studies also reported that the RR genotype is over-represented while the XX genotype is under-represented in strength and power athletes compared to control groups [21, 38, 56].

Our finding that the RR genotype and the R allele of the *ACTN3* R577X polymorphism promote power and speed phenotypes [57] is supported by findings in young male Chinese soldiers where the RR genotype is associated with better anaerobic performance, while those with the XX genotype had lower grip strength [58]. Muscle strength and size are greater for RR and RX genotypes than for XX genotypes [59]. Additionally, other studies show that athletes with the *ACTN3* RR genotype have better sprint times and a higher proportion of type II muscle fibers compared to those with the XX genotype [16, 60]. The percentage and number of IIx fibers are higher in RR-genotype athletes than in XX-genotype athletes, and the α-actinin-3 protein content is higher in type IIx fibers than in type IIa [61]. Finally, athletes with the RR genotype have higher levels of testosterone [16], which may explain, in part, the association between the *ACTN3* RR genotype, skeletal muscle hypertrophy and power athlete status, as athletes carrying the RR genotype are more likely to be power athletes. These observations have led to the *ACTN3* gene being labeled the “speed gene” [14]. Given that the RR genotype is overrepresented in power athletes, it is possible that this genotype augments the chance of being an elite athlete in sports requiring power, speed and strength [19]. The α-actinin-3 protein allows the traction of actin filaments attached to the Z line at high amplitudes of muscle contraction in type II fibers, raising the possibility that athletes with RR and RX genotypes (who express the α-actinin-3) perform better in strength and power activities than those with XX genotypes [36].

There are some findings that do not support the contention that *ACTN3* R577X genotypes are associated with strength and power in weightlifting, powerlifting and throwing events [40]. For example, differences in the distribution of *ACTN3* R577X alleles/genotypes were not observed in a group cohort of athletes or in each group in Russian and Lithuanian weightlifters and throwers [40]. Other studies suggest no benefits of the *ACTN3* R577X polymorphism on muscle strength and other power-based phenotypes [62–66], as also shown when jump heights were measured in volleyball, basketball and rugby players [48, 67].

Collectively, these studies indicate that the *ACTN3* R577X genotype contributes to the likelihood of becoming an elite athlete in aerobic sports but that possessing the XX genotype does not directly negate becoming an elite athlete in these sports disciplines. This is important as the genotyping of the *ACTN3* R577X should not be used to detect talents in anaerobic-sports. The selection of RR athletes in the talent detection process should not occur at the expense of talented athletes with a XX genotype as there may be additional factors to also consider (physical and physiological abilities, psychological attributes, environment and motivation). For physical fitness qualities, the level of inheritance ranges from 20% for (balance) to 70–80% for alactic anaerobic power which is in line with our findings [68]. The practical application of a genotype score to is currently exploratory in nature, and its use in talent prediction is premature and has in fact proven to be ineffective [64–68], likely because the collection of genotypic data from the global population from athletes from different sporting disciplines.

### ACTN3 R577X Polymorphism in Power Athletes Compared to Endurance Athletes

Our findings are in agreement with other studies in which the frequency of the RR genotype and the presence of the R allele was higher in athletes specializing in strength-power sports [21]. Additionally, the frequency of the RR genotype was higher in sprint/power, martial arts, and ball game sports athletes [46]. These findings suggest a crucial role for α-actinin-3, which is expressed only in individuals with the R-allele, in elite level sporting activities with a large anaerobic component. A study of top Finish sprinters demonstrated none of them possessed the XX genotype [69] Soccer players with the XX genotype showed higher VO_2_ peak levels than their RR genotype counterparts [45]. Conversely, individuals with the RR genotype showed reduced times in 10, 20 and 30m sprints, as well as better performance in jumping tests [45]. The XX genotype is underrepresented in Russian endurance athletes [70], which may indicate that in addition to endurance capacity, power and speed components are also necessary for success in endurance sports [71–73]. On the other hand, data from Asia and Africa suggests that a deficiency of *ACTN3* is not related to endurance performance [74, 75]. Athletes with the RR genotype have higher explosive leg muscle power in jump tests compared to those with the XX genotype, but some studies report no difference between RX and XX genotypes [9].

In contrast, some studies reported that homozygous XX athletes had higher explosive power than athletes with a RR genotype [67], or that was no association between the *ACTN3* gene and power/strength phenotypic traits [50]. The latter study had a limitation because the tests were not specific to the activity. *ACTN3* knockout mice show improved endurance performance, characterized by high oxidative enzyme activity and a transition to slow-twitch type I muscle fibers [76, 77], which are highly vascularized and contain many mitochondria [78]. A deficiency of the XX genotype leads to a lack of α-actinin-3 protein, with reductions in athletic power and speed [19]. Additionally, the changes in type II muscle fibers due to the absence of *ACTN3* reduces sprint performance and improves endurance performance in athletes with a XX genotype [21, 69]. Individuals with the XX genotype have superior physical characteristics compared to those with the R allele, due to a higher proportion of Type I fibers [77], a higher ventilation threshold respiratory compensation point [79], a higher fatigue index [80], decreased glycogen depletion in type II fibers after exercise [81], and a higher VO_2peak_ [82]. Moreover, the proportion of subjects with aerobic energy metabolism for maximal and submaximal exercise was high for the athletes with a XX genotype [77, 79, 82].

### ACTN3 R577X Polymorphism in Male and Female Power Athletes

Athletic performance can be influenced by sex-specific genetic factors such as differences in the physical, physiological and psychological abilities, including factors specific to males and females [83, 84]. Our meta-analysis shows that the RX genotype is slightly more frequent in male than in female power athletes (*p* = 0.06), while no differences were observed for the expression of RR and XX genotypes. In contrast to our findings, the distribution of RX was reported by others to be greater in female than in male sprinters (57% versus 39%) [85]. Yang et al. [12] found female sprint/power athletes lacked the XX genotype. Peak power was greater in male athletes with the R dominant allele compared to the XX group, but this difference was not present in female athletes [86]. Additionally, there is a higher frequency of the R allele in elite female sprint swimmers than in national level female swimmers and controls [87]. However, the *ACTN3* XX genotype was similarly under-represented in both male and female artistic gymnasts compared to controls [54]. Female weightlifters have a higher ratio of the XX genotype (16% in females compared to 14% in males) [51]. On the other hand, no significant difference between sexes were observed in the frequency of genotypes between those with the best and worst performance in terms of power [66]. A study by Clarkson et al. [88] reported no correlation between *ACTN3* R577X polymorphisms and isometric elbow flexion strength in males, although females with the XX genotype had lower isometric strength than those with a heterozygous RX. A study by Orysiak et al. [50] found no association between sports disciplines and *ACTN3* genotype variants for female and male athletes, and the distribution of *ACTN3* R577X polymorphisms did not differ between male and female athletes. Some studies have reported that the absence of the actinin-3 protein negatively influences the maximal isokinetic knee extension torque in middle-aged women, but not in males [89]. However, as genotypes can have different effects on athletic performance in female and male athletes, gene expression may be constructed differently for each sex [90].

### Strengths and Limitations

This study expands on previous studies on the association between *ACTN3* R577X polymorphisms and performance in sports of a short and intense nature. Our meta-analysis included 25 eligible studies conducted in 13 different countries with controls and athletes from different specialties. An assessment of the association between *ACTN3* R577X polymorphisms and anaerobic exercise in various sports (power athletes, power athletes vs. controls, power athletes vs. endurance athletes, and male vs. female power athletes) indicates that the RR genotype and the R allele has greater benefits in power athletes, while the XX genotype and the X allele may be less favorable. Our meta-analyses were strengthened by a low heterogeneity in the studies, where seven forest plots indicated low heterogeneity (< 50%) and five forest plots demonstrated moderate heterogeneity (< 70%).

Our systematic review and meta-analysis has some limitations: (1) we identified only five studies that included both sexes with relatively small total sample sizes of males (n = 564) and females (n = 296), suggesting caution in discussing the role of sex on the distribution of *ACTN3* R577X genotypes in power athletes; (2) although this study analyzed a large number of eligible studies (25 studies) with a large total sample size (14,541 participants), more studies are needed to support any recommendations, particularly related to the future use of genetic data in the orientation, optimization, adaptation of training and planning of the workload of athletes.

## Conclusions

Our systematic review with meta-analysis provides additional evidence to support the notion that genetic variations contribute to athletic status and sports performance. Findings of this study suggest that the frequency of the *ACTN3* R577X genotypes in power athletes followed a RX > RR > XX pattern, with a dominance of the R allele over the X allele. However, the RR genotype was overrepresented in power athletes compared non-athletes and endurance athletes, while the XX was under-represented in power athletes. Collectively, the study results suggest that the RR genotype, associated with a normal expression of α-actinin-3 in fast-twitch fibers, could be useful to elite performers of anaerobic-based exercise, and can increase athletic abilities in short and highly intense sports.

From a practical perspective, *ACTN3* genotyping in athletes may allow coaches to better tailor training programs in terms of frequency and intensity to the athlete's genetic constitution and optimize performance and reduce the likelihood of injury. For example, an athlete with an RR genotype could benefit from strength and power training to improve vertical/horizontal jump performances and linear as well as change-of-direction speed, while an athlete with an XX genotype could benefit from endurance training to improve aerobic capacity. However, the type of training more suitable for each athlete according to their *ACTN3* genotype is still under debate and further research is warranted in this topic. In any case, genotyping should not be used to select those athletes who could have a higher probability of becoming elite for either power or endurance sports, as this would introduce selection bias [91]. Regarding future studies, we propose a comprehensive exploration of the *ACTN3* gene and various biomarkers in elite athletes from various sporting disciplines. This approach aims to elucidate the physiological implications of the *ACTN3* R577X polymorphism and its potential influence on athletic performance.

## Supplementary Information

**Additional file 1:** The funnel plots for the comparison of genotype frequencies:** Figure S1.** Funnel plot of ACTN3 R577X polymorphism in power athletes (RR vs. RX genotypes).** Figure S2.** Funnel plot of ACTN3 R577X polymorphism in power athletes (RR vs. XX genotypes).** Figure S3.** Funnel plot of ACTN3 R577X polymorphism in power athletes (RX vs. XX genotypes).** Figure S4.** Funnel plot of ACTN3 R577X polymorphism in power athletes (R vs. X alleles).** Figure S5.** Funnel plot of RR genotype expression in power athletes versus controls.** Figure S6.** Funnel plot of RX genotype expression in power athletes versus controls.** Figure S7.** Funnel plot of XX genotype expression in power athletes versus controls. **Figure S8.** Funnel plot of R allele in power athletes versus controls.** Figure S9.** Funnel plot of X allele in power athletes versus controls.** Figure S10.** Funnel plot of RR genotype expression in power versus endurance athletes.** Figure S11.** Funnel plot of RX genotype expression in power versus endurance athletes. **Figure S12.** Funnel plot of XX genotype expression in power versus endurance athletes. **Figure S13.** Funnel plot of R allele in power athletes versus endurance athletes. **Figure S14.** Funnel plot of X allele in power athletes versus endurance athletes.

## Data Availability

All data supporting the findings of this study are available in this published article.
