# Supplementary material for: A Systematic Review and Meta-analysis of the Association Between ACTN3 R577X Genotypes and Performance in Endurance Versus Power Athletes and Non-athletes
Source: Sports Med Open. 2024 Apr 12;10:37. doi: 10.1186/s40798-024-00711-x (PMC11014841; doi:10.1186/s40798-024-00711-x)
Supplement: Supplementary file 1 — Additional file 1: The funnel plots for the comparison of genotype frequencies: Figure S1. Funnel plot of ACTN3 R577X polymorphism in power athletes (RR vs. RX genotypes). Figure S2. Funnel plot of ACTN3 R577X polymorphism in power athletes (RR vs. XX genotypes). Figure S3. Funnel plot of ACTN3 R577X polymorphism in power athletes (RX vs. XX genotypes). Figure S4. Funnel plot of ACTN3 R577X polymorphism in power athletes (R vs. X alleles). Figure S5. Funnel plot of RR genotype expression in power athletes versus controls. Figure S6. Funnel plot of RX genotype expression in power athletes versus controls. Figure S7. Funnel plot of XX genotype expression in power athletes versus controls. Figure S8. Funnel plot of R allele in power athletes versus controls. Figure S9. Funnel plot of X allele in power athletes versus controls. Figure S10. Funnel plot of RR genotype expression in power versus endurance athletes. Figure S11. Funnel plot of RX genotype expression in power versus endurance athletes. Figure S12. Funnel plot of XX genotype expression in power versus endurance athletes. Figure S13. Funnel plot of R allele in power athletes versus endurance athletes. Figure S14. Funnel plot of X allele in power athletes versus endurance athletes. [file 40798_2024_711_MOESM1_ESM.docx]

Supplementary Material

**Funnel plots**


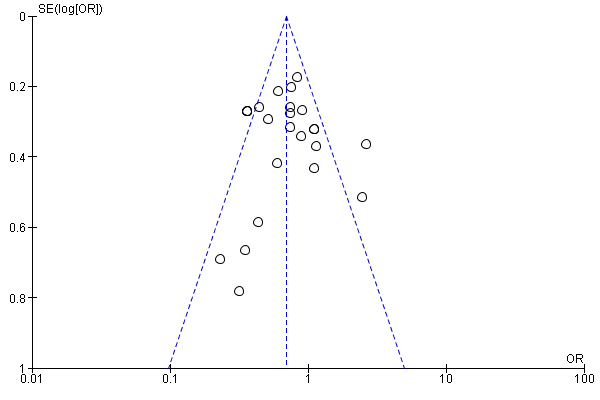


**Figure S1:** Funnel plot of ACTN3 R577X polymorphism in power athletes (RR vs. RX genotypes).


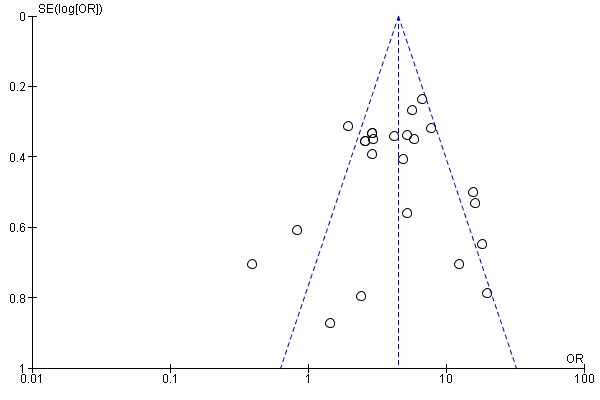


**Figure S2:** Funnel plot of ACTN3 R577X polymorphism in power athletes (RR vs. XX genotypes).


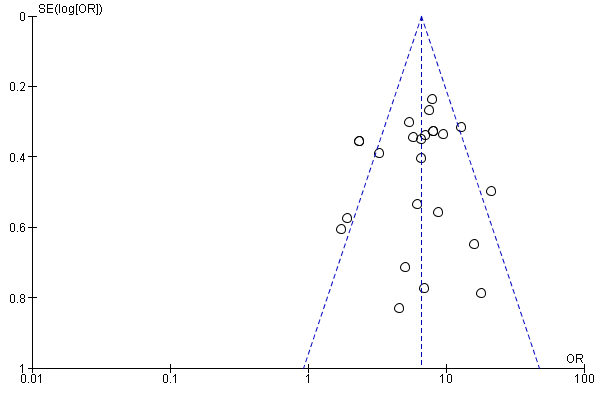


**Figure S3:** Funnel plot of ACTN3 R577X polymorphism in power athletes (RX vs. XX genotypes).


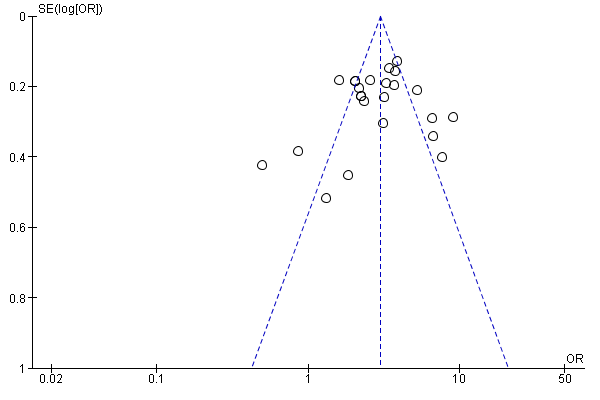


**Figure S4:** Funnel plot of ACTN3 R577X polymorphism in power athletes (R vs. X alleles).


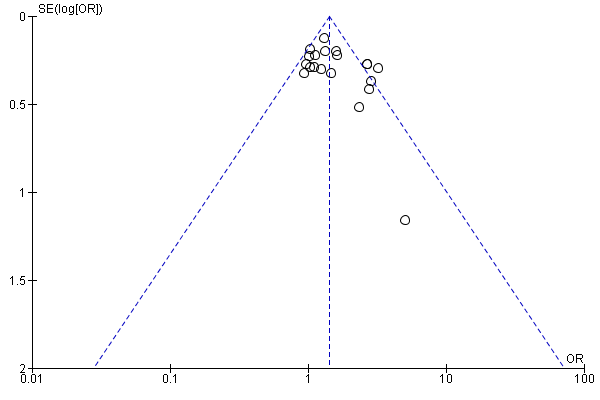


**Figure S5:** Funnel plot of RR genotype expression in power athletes versus controls.


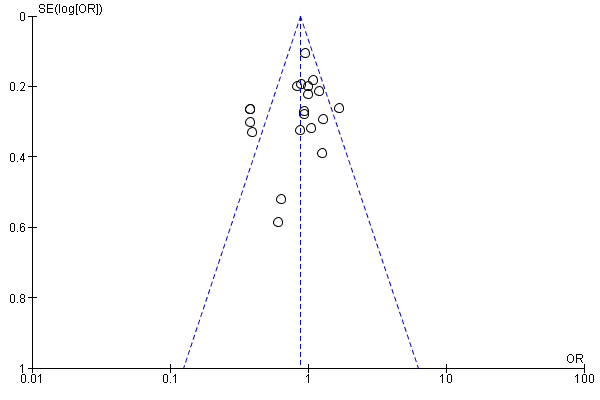


**Figure S6:** Funnel plot of RX genotype expression in power athletes versus controls.


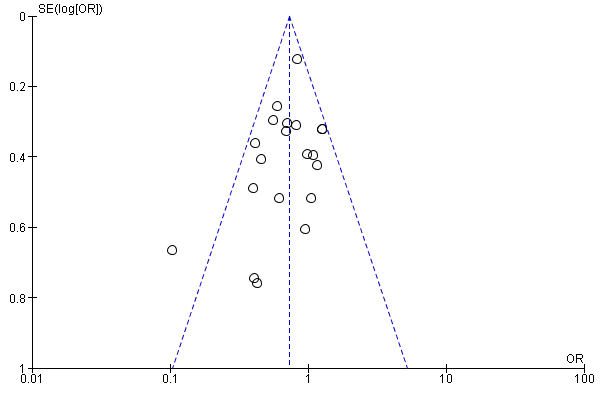


**Figure S7:** Funnel plot of XX genotype expression in power athletes versus controls.


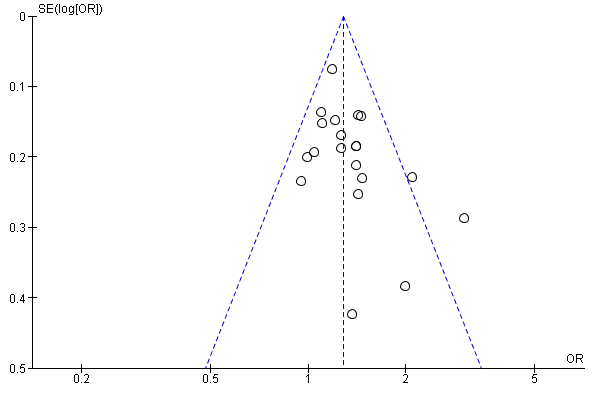


**Figure S8:** Funnel plot of R allele in power athletes versus controls.


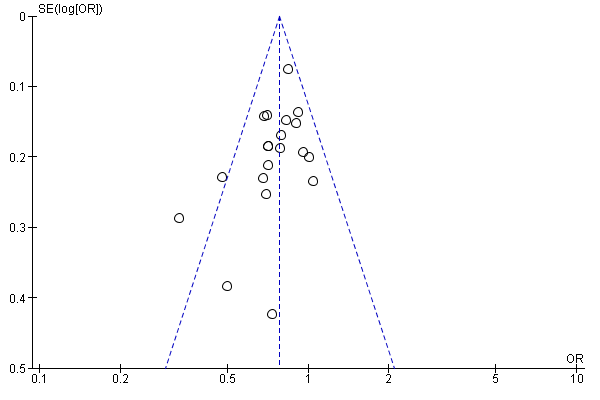


**Figure S9:** Funnel plot of X allele in power athletes versus controls.


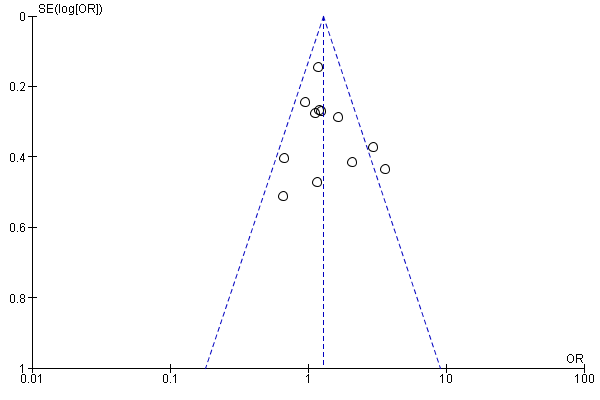


**Figure S10:** Funnel plot of RR genotype expression in power versus endurance athletes.


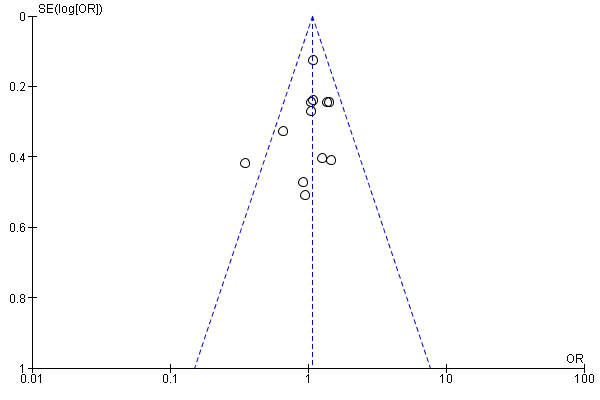


**Figure S11:** Funnel plot of RX genotype expression in power versus endurance athletes.


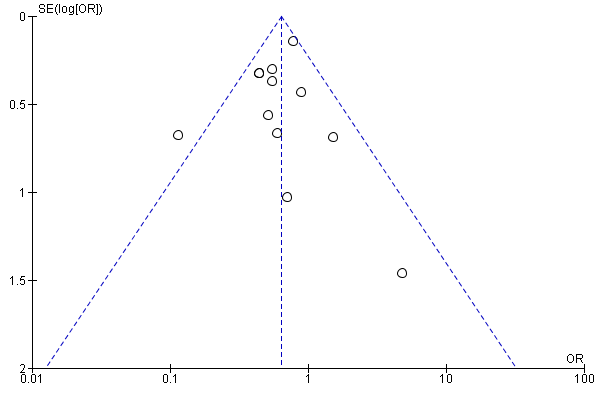


**Figure S12:** Funnel plot of XX genotype expression in power versus endurance athletes.


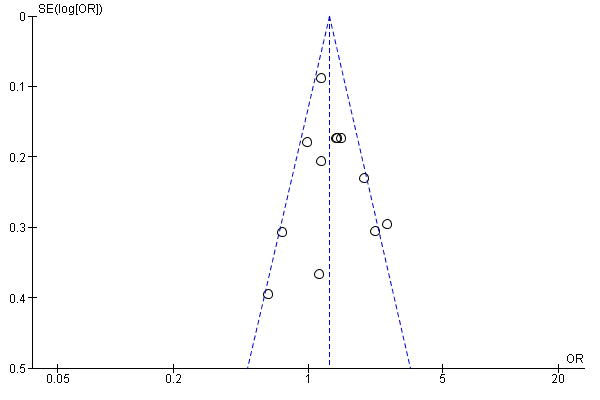


**Figure S13:** Funnel plot of R allele in power athletes versus endurance athletes.


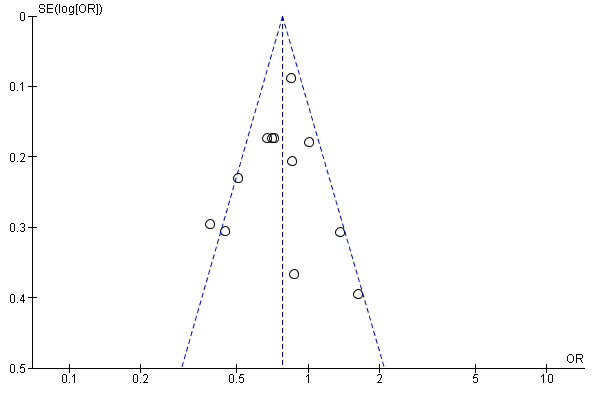


**Figure S14:** Funnel plot of X allele in power athletes versus endurance athletes.
